# 286. Prevalence and Clinical Outcomes of COVID-Associated Pulmonary Fibrosis

**DOI:** 10.1093/ofid/ofac492.364

**Published:** 2022-12-15

**Authors:** Andrew J Vogler, Anselmo Garcia, Neema Mafi, Chad M VanDenBerg, Vanthida Huang

**Affiliations:** Midwestern University College of Pharmacy-Glendale Campus, Glendale, Arizona; Arizona Critical Care Specialists (AZCCS), HonorHealth John C. Lincoln Medical Center, Phoenix, Arizona; Apex Physicians Group, HonorHealth John C. Lincoln Medical Center, Phoenix, Arizona; Midwestern University Clinical Research Services, Glendale, Arizona; Midwestern University College of Pharmacy-Glendale Campus, Glendale, Arizona

## Abstract

**Background:**

Pulmonary fibrosis (PF) is a well-known consequence of severe lung disease and is associated with permanent changes as well as irreversible pulmonary dysfunction. The development of PF in patients infected with COVID-19 has been documented in multiple studies and case reports. However, prevalence and outcomes associated with PF have not been well established. Therefore, we sought to evaluate the prevalence and clinical outcomes of PF among patients infected with COVID-19.

**Methods:**

This is an observational cohort study from January 2020 – January 2022. We collected data from adult patients diagnosed with COVID-19 who had at least two separate computerized tomography (CT) scans. Patients were grouped based on whether evidence of developing PF, defined by clinical radiological parameters, were seen on CT. The initial CT must be negative for PF to be included. We collected the following information: baseline characteristics, secondary infection, mortality, and treatments. The primary objective was to determine the prevalence of PF among COVID-19 patients. Secondary objectives were to evaluate the differences in 30-day all-cause mortality; intensive care unit (ICU) mortality; and prevalence of secondary infections.

**Results:**

A total 161 patients were COVID-19 positive with multiple CT scans; 27 (16.8%) had signs of PF while 134 (83.2%) did not. Of the patients with signs of PF, 13 (48.1%) were male (mean age of 57 years) and 13 (48.1%) were admitted to the ICU. There was no difference in secondary bacterial infection between fibrotic and non-fibrotic patients (55.6% vs 38.5%, p=0.20). The most common bacterial infection among PF patients was caused by *S. aureus* (26.7%). The most common infection type among PF patients was nosocomial pneumonia (46.7%). Mortality at 30-day was higher in the fibrotic patients, 70% vs 24.2% (p< 0.01). The ICU mortality trended higher in the fibrotic group (69.2% vs 36.6%, p=0.124), although it was not statistically significant.

**Conclusion:**

Pulmonary fibrosis can be fatal as a complication of COVID-19. Further investigation is warranted to evaluate the outcomes of PF in patients with COVID-19.

**Disclosures:**

**All Authors**: No reported disclosures.

